# Characteristics of candidate genes associated with embryonic development in the cow: Evidence for a role for *WBP1* in development to the blastocyst stage

**DOI:** 10.1371/journal.pone.0178041

**Published:** 2017-05-18

**Authors:** M. Sofia Ortega, Justin J. Kurian, Robert McKenna, Peter J. Hansen

**Affiliations:** 1 Department of Animal Sciences, D.H. Barron Reproductive and Perinatal Biology Research Program and Genetics Institute, University of Florida, Gainesville, Florida, United States of America; 2 Department of Biochemistry and Molecular Biology, University of Florida, Gainesville, Florida, United States of America; Michigan State University, UNITED STATES

## Abstract

The goal was to gain understanding of how 12 genes containing SNP previously related to embryo competence to become a blastocyst (*BRINP3*, *C1QB*, *HSPA1L*, *IRF9*, *MON1B*, *PARM1*, *PCCB*, *PMM2*, *SLC18A2*, *TBC1D24*, *TTLL3* and *WBP1*) participate in embryonic development. Gene expression was evaluated in matured oocytes and embryos. *BRINP3* and *C1QB* were not detected at any stage. For most other genes, transcript abundance declined as the embryo developed to the blastocyst stage. Exceptions were for *PARM1* and *WBP1*, where steady-state mRNA increased at the 9–16 cell stage. The SNP in *WBP1* caused large differences in the predicted three-dimensional structure of the protein while the SNP in *PARM1* caused smaller changes. The mutation in *WBP1* causes an amino acid substitution located close to a P-P-X-Y motif involved in protein-protein interactions. Moreover, the observation that the reference allele varies between mammalian species indicates that the locus has not been conserved during mammalian evolution. Knockdown of mRNA for *WBP1* decreased the percent of putative zygotes becoming blastocysts and reduced the number of trophectoderm cells and immunoreactive CDX2 in the resulting blastocysts. *WBP1* is an important gene for embryonic development in the cow. Further research to identify how the SNP in *WBP1* affects processes leading to differentiation of the embryo into TE and ICM lineages is warranted.

## Introduction

During the preimplantation period, the mammalian embryo undergoes a series of morphological, molecular, physiological and metabolic processes that transform a single-cell totipotent zygote into a multicellular blastocyst composed of pluripotent inner cell mass and differentiated trophectoderm [1]. Initially, the embryonic genome is quiescent and the embryo relies on maternally-derived mRNA stored in the oocyte for new protein synthesis [2]. Major activation of the embryonic genome occurs at a species-dependent time in development, occurring at the two-cell stage in the mouse [3], at the four to eight cell-stage in the human [4, 5], and at the eight-cell stage in the bovine [6]. Not surprisingly, given the myriad of molecular and cellular events required for successful development, the mammalian embryo often fails to successfully develop to the blastocyst stage. In the cow, the species studied here, it has been estimated that 31–47% percent of fertilized embryos are not viable by day 6 of development [7, 8]. Incidence of embryonic mortality can increase under various physiological conditions including lactation and undernutrition [9–11].

Several allelic variants in specific genes have been identified that are associated with development of the bovine embryo to the blastocyst stage *in vitro*. These include single nucleotide polymorphisms (SNP) in genes involved in extracellular ligand signaling (*BMP4*, *BRINP3*, *FGF2*, *STAT5A*, *TBC1D24* and *WBP1*), endo- and exocytosis (*MON1B*, *PMM2*, *SLC18A2*, *TTLL3*), regulation of apoptosis (*PARM1*), protection from cellular stress (*HSPA1L)*, energy metabolism (*PCCB*), protein-protein interactions (*C1QB*, *WBP1*) and transcriptional regulation *(IRF9*) [12–15].

Little is known about how allelic variants in genes act to modify the prospects of an embryo for successful development. Of the 12 SNP found by Cochran *et al*. [15] to be associated with development of embryos to the blastocyst stage, one is in the regulatory region of *HSPA1L* and affects gene transcription [16], but the other 11 (*BRINP3*, *C1QB*, *IRF9*, *MON1B*, *PARM1*, *PCCB*, *PMM2*, *SLC18A2*, *TBC1D24*, *TTLL3* and *WBP1*) cause a change in amino acid in the sequence. It is likely that many genes affect development at specific stages because expression is limited to specific periods of development. In the bovine, for example, transcription first occurred for 390 genes at the four-cell stage, 3,965 genes at the eight-cell stage, 628 genes at the 16-cell stage, and 1,865 genes at the blastocyst stage [17].

The goal of the present experiment was to gain understanding of how the 12 genes found by Cochran *et al*. [15] which contain allelic variants associated with development could modify function of the preimplantation embryo. In the experiment of Cochran *et al*. [15], oocytes were fertilized with sires of known genotypes and effect of sire genotype on subsequent development was determined. With this experimental design, a SNP could have either directly affected embryonic development, when the paternal allele becomes expressed during development, or a SNP could have affected development indirectly by affecting sperm function. This is so because ability of sperm for fertilization can affect competence of the resultant embryo for development [18].

The first objective of the present series of experiments was to evaluate temporal changes in expression of the 12 genes to understand the period during development when the gene is active. A finding that the gene is expressed coincident with or after the major round of embryonic genome activation at the 8-cell stage is consistent with direct effects of the SNP on embryonic development. Conversely, if the gene is not expressed in the embryonic period, associations of the SNP with development likely represent indirect effects on the sperm. A second objective was to model how changes in amino acid sequence caused by the SNP changes tertiary structure of the protein for those genes whose expression was increased at the time of embryonic genome activation. Significant changes in protein structure would be indicative that the SNP could change the function of the protein in the embryo. Finally, an experiment was performed to test whether one gene implicated in genetic variation in embryonic development, *WBP1*, is necessary for embryonic development. *WBP1* contains a SNP that has been associated with cow conception rate [19] as well as competence of embryos to develop to the blastocyst stage *in vitro* [15]. Nothing is known about the role of WBP1 in embryonic development. However, WBP1 binds to the WW domain of a variety of proteins including the transcription factor YAP [20] that is required for formation of trophectoderm (TE) in the blastocyst [21]. It was hypothesized that reduction in mRNA abundance for WBP1 would reduce development to the blastocyst stage and formation of cells in the inner cell mass (ICM) and TE.

## Materials and methods

### Developmental changes in gene expression

#### Embryo production

Ovaries were obtained from Central Packing Co. (Center Hill, FL, USA) from cattle of *Bos taurus* and admixtures of *B*. *taurus* and *B*. *indicus*. The surface of each ovary was sliced with a scalpel to harvest immature cumulus-oocyte complexes (COC) into oocyte collection medium (composition of all media is presented in S1 Table). The COC were washed and groups of 10 were matured in 50 μL droplets of oocyte maturation medium covered with mineral oil (Sigma, St. Louis, MO) for a period of 21 h at 38.5°C in a humidified atmosphere of 5% (v/v) CO_2_. For each replicate, up to 300 matured COCs were washed three times in a medium called HEPES-SOF before being placed in a 35 mm dish containing 1.7 ml of SOF-FERT. Insemination of each replicate of fertilization was performed with semen pooled from three individual bulls of various breeds (the total number of bulls in the experiment were 17). Sperm were purified from frozen-thawed straws of extended semen using a Percoll gradient [45% (v/v) and 90% (v/v) Percoll] and diluted in SOF-FERT to achieve a final concentration of 1x10^6^/ml in the fertilization dish. In addition, 80 μL PHE solution was added to improve sperm motility and promote fertilization. Fertilization proceeded for 8–9 h at 38.5°C in a humidified atmosphere of 5% (v/v) CO_2._ Putative zygotes (i.e., oocytes exposed to sperm) were denuded from the surrounding cumulus cells at the end of fertilization by vortexing groups of 200–300 putative zygotes for 5 min in 600 μL of HEPES-SOF containing 10,000 U/ml hyaluronidase. Embryos were then cultured in groups of 30 in 50 μl drops of culture medium (SOF-BE2) covered with mineral oil at 38.5°C in a humidified atmosphere of 5% (v/v) O_2_ and 5% (v/v) CO_2_ with the balance N_2_, until the moment of collection.

Pools of 40 matured oocytes or embryos were collected. Matured oocytes were collected after 21 h of maturation. Embryos were collected at the 2-cell [27–31 h post insemination (hpi)], 3–4 cell (46–52 hpi), 5–8 cell (49–59 hpi), 9–16 cell (72–75 hpi), morula (120–123 hpi) and blastocyst (168–171 hpi) stages. A total of 5 pools were analyzed for each of the 7 stages.

#### Reverse transcription (RT) and quantitative PCR

Analysis of gene expression was accomplished by quantitative RT- PCR. Briefly, pools of embryos were treated with 0.1% (w/v) protease from *Streptococcus griseus* to remove the zona pellucida, washed three times in 50 μl droplets of Dulbecco’s phosphate buffered saline containing 1% (w/v) polyvinylpyrrolidone (DPBS-PVP), placed in 100 μl extraction buffer from the PicoPure^®^ RNA isolation kit (Applied Biosystems, Carlsbad, CA, USA), and kept at -80°C so that processing of each stage could be performed at one time. Total RNA was isolated using the PicoPure^®^ RNA isolation kit (Applied Biosystems) following the manufacturer’s instructions. RNA was treated with 1 μL (2 U) of DNAse (New England Biolabs, Ipswich, MA, USA) per sample, and then reverse-transcribed using the High Capacity cDNA Reverse Transcription Kit^®^ (Applied Biosystems) to produce complementary DNA (cDNA). From each sample, a negative control was produced by incubation without reverse transcriptase. The cDNA was stored at -20°C until further use.

PCR was performed using a CFX96 Real-Time PCR detection System (Bio-Rad, Hercules, CA, USA) and the SsoFast EvaGreen Supermix^®^ with Low ROX (Bio-Rad). Each reaction contained 1 μl forward primer (0.5 mM), 1 μl reverse primer (0.5 mM), 12 μl Evagreen Supermix (Bio-Rad), 6.8 μl H_2_O and 1.2 μl of cDNA sample; all samples were run in duplicate. Amplification conditions were: 95°C for 30 sec, 40 cycles at 95°C for 5 sec, 60°C for 5 sec, and 1 cycle of melt curve analysis at 65–95°C in increments of 0.5°C every 2 sec.

Primer sequences are detailed in S2 Table. The sequence for *HSPA1L* was obtained from the literature [22]. For the remaining genes, primers were designed using the Primer Quest^®^ tool from Integrated DNA Technologies (Coralville, IA, USA). Each set of primers was validated by performing a standard curve, where the cycle threshold (CT) values of five serial dilutions were subjected to regression versus the log of input nucleic acid. The slope of the regression was estimated, and only primers that yielded a slope between -3 and -3.3, corresponding to primer efficiency of 100–110%, were used. Moreover, melting curves were evaluated to ensure that a single, specific product was generated. Amplicon size was evaluated by agarose gel electrophoresis. The PCR product was subjected to Sanger sequencing and the sequence confirmed by using the Basic Local Alignment Search Tool (BLAST) from the National Center for Biotechnology Information (NCBI).

#### Statistical analysis

The Δ cycle threshold (Δ CT) was determined by subtracting the average CT value of the sample from the geometric mean of the CT for three housekeeping genes, *SDHA*, *GAPDH* and *YWHAZ* [23, 24]. Fold change was calculated relative to the housekeeping genes (2 ^ΔCT^). Stage of development effects on gene expression were analyzed by least-squares analysis of variance of the ΔCT values using the GLM procedure of the Statistical Analysis System SAS version 9.4 (SAS Institute Inc., Cary, NC, USA). Differences among means were evaluated by the pdiff option of the LSMEANS statement. Replicate and stage were included as main effects in the model.

### Protein structure prediction

Amino acid sequences were retrieved from the NCBI database. Predicted protein structures for both allelic variants of PARM1 (NP_001069239.1) and WBP1 (NP_001029518.1) were determined using the I-Tasser v4.4 software program [25]. The program pipeline consists of four general phases: threading template identification through a non-redundant structure library to identify templates, iterative structure assembly simulation, model selection and refinement, and structure-based function annotation [25–27]. The three-dimensional models for each variant of the protein were superimposed and aligned so as to identify potential changes in structure. Visualization of the models, location of the amino acid substitution, and alignment of the models was performed using the software PyMOL Molecular Graphics System Version 1.8 (Schrödinger, Cambridge, MA, USA).

### Phylogenetic analysis

The SNP in *WBP1* was subjected to phylogenetic analysis. The coding sequence was retrieved from the NCBI nucleotide database [28] and sequences for other mammalian organisms available in the database were obtained using the BLAST tool. The number of species was 50. Sequences were aligned using the software CLUSTAL Omega [29]. Aligned sequences were used for phylogenetic and molecular evolutionary analyses conducted using MEGA version 6 [30]. The evolutionary history was inferred using the Maximum Likelihood method based on the Kimura 2-parameter model [31, 32]. Bootstrap values were calculated based on 1000 replicates to assess the level of confidence of each branch pattern [33]. Initial trees from heuristic search were obtained automatically by applying Neighbor-Join and BioNJ algorithms to a matrix of pairwise distances estimated using the Maximum Composite Likelihood (MCL) approach, and then by selecting the topology with superior log likelihood value. A discrete gamma distribution was used to model evolutionary rate differences among sites (5 categories (+G, parameter = 0.5809)). All position containing gaps and missing data were eliminated [28]. Subsequently, the nucleotide at the SNP in the bovine gene related to embryonic development was identified for each species and mapped to the phylogenetic tree.

### Knockdown of *WBP1* during preimplantation development

Knockdown was performed using GapmeR LNA^™^ antisense oligonucleotides from Exiqon (Woburn, MA, USA). The GapmeR LNA^™^ are nucleotide analogues in which the ribose ring is locked in an N-type conformation so that when incorporated to a RNA sequence of interest, the binding affinity towards complementary RNA is increased to optimize activation of RNase H and degradation of the target mRNA [34, 35]. Incorporation into the cells involves multivesicular bodies and endosomal trafficking [36, 37]. Two GapmeR were designed: one to target the *WBP1* sequence and a scrambled version of the same sequence used as a negative control. The sequence that targeted *WBP1* (termed anti-WBP1) was: 5’- AGGCGAAGGTCAAGCA -3’. The sequence for the scrambled negative control was 5’-GCAGCGTACAAGGAAG-3’.

Embryos were produced in vitro as indicated above except that oocyte washing medium (MOFA Global, Verona, WI) was used instead of OCM; HEPES-TALP (Tyrode’s albumin lactate pyruvate) was used instead of HEPES-SOF, and IVF-TALP was used instead of SOF-FERT (composition of media is detailed in S1 Table). The experiment was replicated 5 times. For each replicate, embryos were treated at 20–22 hpi with 5 μM anti-WBP1 GapmeR, 5 μM scrambled GapmeR, or vehicle added to the culture medium. At 72–75 hpi, groups of 18–20 9–16 cell embryos per replicate were collected from each treatment to evaluate *WBP1* expression by quantitative PCR following procedures described above. Amounts of mRNA for *WBP1* were expressed as the fold change relative to the vehicle control group (2 ^ΔΔCT^).

The remaining embryos (232 embryos for vehicle, 197 for scrambled control and 214 for anti-*WBP1* GapmeR treatment across the five replicates) were cultured until Day 8 after insemination. Cleavage was assessed at Day 3 and blastocyst formation at Day 7 and 8. Blastocysts were collected at Day 8 to determine inner cell mass and trophectoderm cell number by determining nuclear CDX2. Blastocysts were washed three times in DPBS-PVP, fixed in 4% (w/v) paraformaldehyde in DBPS/PVP for 15 min, washed in DPBS-PVP, incubated in permeabilization solution [DPBS containing 0.5% (v/v) Triton X-100] for 30 min, and then incubated for 1 h in blocking buffer [DPBS containing 5% (w/v) bovine serum albumin (BSA)]. Blastocysts were incubated overnight (4°C) with mouse anti-mouse monoclonal CDX2 antibody, ready to use (Biogenex, Fremont, CA, USA). After incubation, blastocysts were washed 6 times in wash buffer [DPBS containing 0.1% Tween 20 and 0.1% (w/v) BSA], and incubated with 1 μg/ml goat anti-mouse IgG conjugated with fluorescein isothiocyanate (FITC; Abcam, Cambridge, MA, USA) in the dark. Blastocysts were again washed 6 times in wash buffer. Nuclear labeling was achieved using Hoechst 33342 (1 μg/ml in DPBS-PVP) for 15 min in the dark. Lastly, blastocysts were rinsed in DPBS-PVP and placed on a slide containing 1 drop of SlowFade Gold antifade reagent (Life Technologies, Carlsbad, CA, USA), covered with a coverslip, and observed with a X40 objective using a Zeiss Axioplan 2 epifluorescence microscope (Zeiss, Göttingen, Germany) and Zeiss filter sets 02 [4’,6’-diamidino-2- phenylindole (DAPI)] and 03 (FITC). Digital images were acquired using AxioVision software (Zeiss) and a high-resolution black and white Zeiss AxioCam MRm digital camera. For control groups, the primary antibodies were replaced with IgG from the species in which the primary antibody was raised. Total cell number was determined by counting nuclei labeled with Hoescht 33342. The total number of blastocysts subjected to immunofluorescent labeling was 37, 32 and 36 for vehicle, scrambled control and anti-*WBP1* GapmeR treatment.

Analysis of the images was performed using ImageJ V. 1.48 (National Institutes of Health, Bethesda, MD). Trophectoderm cell number was determined by counting nuclei labeled for CDX2 and ICM cell number was estimated as the difference between total and TE cell number. In addition, fluorescent intensity for nuclear CDX2 was determined. The procedure was performed by manually drawing a boundary around each CDX2-positive nucleus, obtaining average pixel intensity in the encircled area and subtracting the background intensity obtained from a region of the image not containing the embryo.

Treatment effects on expression of *WBP1* were analyzed by least-squares analysis of variance of ΔCT using the GLM procedure of SAS. Treatment effects on embryonic development were analyzed by logistic regression using the GENMOD procedure, and mean separation was performed by the pdiff option of the LSMEANS procedure of SAS. Each embryo was considered as an observation. Replicate was considered random and treatment fixed. Treatment effects on cell number and fluorescent intensity were analyzed by least-squares analysis of variance using the MIXED procedure of SAS. In addition contrasts were used to determine differences among treatments. The model included replicate as a random effect and treatment as a fixed effect.

## Results

### Gene expression

Temporal changes in gene expression were assessed to understand the period during development when the gene is expressed. Transcripts were detected for 10 of the 12 genes evaluated; *BRINP3* and *C1QB* were not detected at any stage. Of the 10 genes that were detectable, there was a wide range of transcript abundance. The most highly expressed genes were *HSPA1L*, *PMM2* and *TBC1D24*. Transcript abundance for *HSPA1L* was greater than the geometric mean of housekeeping genes from the oocyte to the 3–4 cell stage of development (i.e., the fold-change relative to housekeeping genes was > 1.0). The fold-change relative to housekeeping genes for *PMM2* and *TBC1D24* was as high as 1.03 and 1.02, respectively. For the remainder of the genes, transcript abundance was lower, with fold-change relative to the housekeeping genes being no higher than 0.04–0.26.

**Fig 1:**
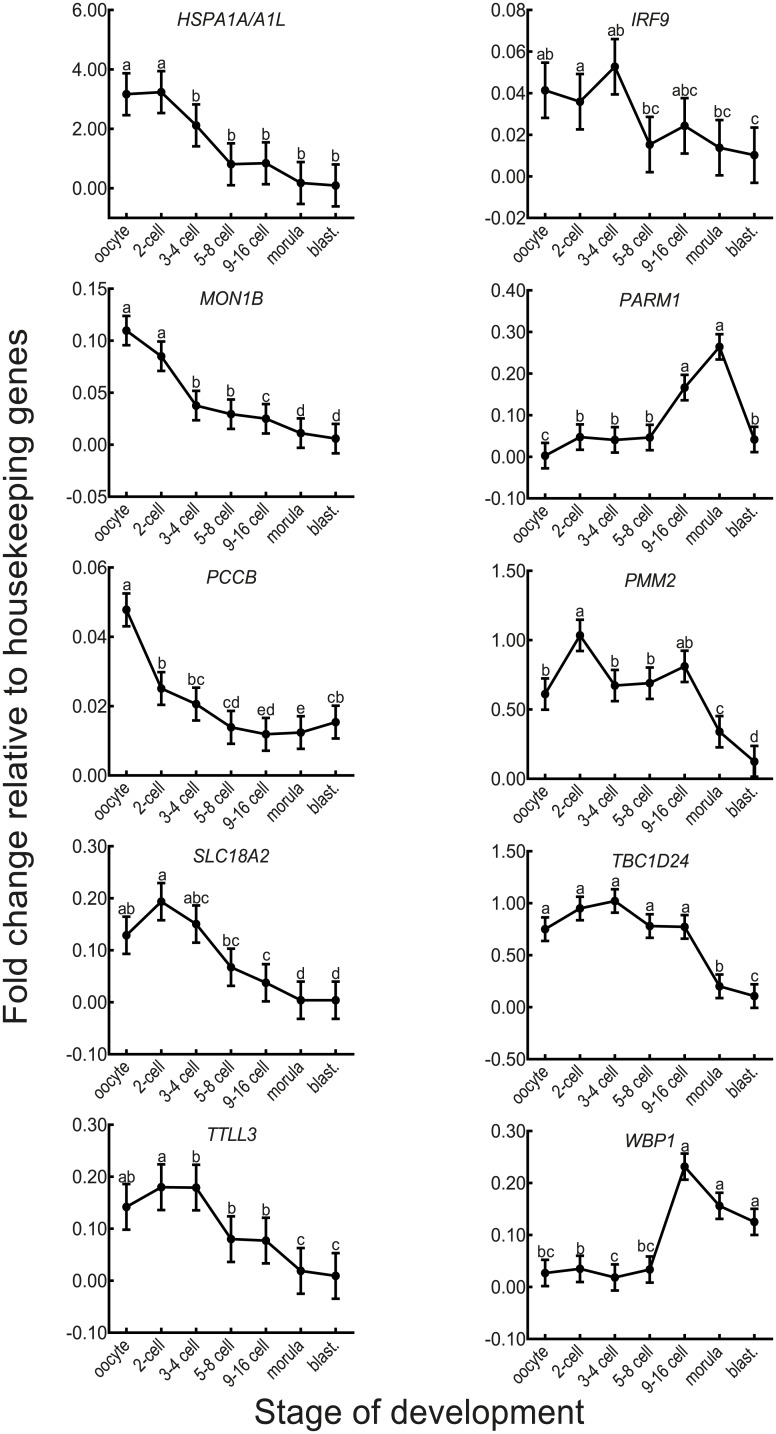
Patterns of expression of genes during preimplantation period. Data are least-squares means ± SEM of fold-change values relative to the geometric mean of three housekeeping genes. Stage of development affected gene expression for *IRF9* (P<0.001) and other genes (P<0.0001). blast.: blastocyst. Means with different superscripts differ from each other (P<0.05).

Effect of stage of development on transcript abundance for each of the detectable genes is illustrated in Fig 1. For each gene, transcript abundance was affected by stage of development (P<0.001 for *IRF9* and P<0.0001 for other genes). For most genes, there was a decline in transcript abundance as the embryo developed to the blastocyst stage. Relative to transcript abundance in the oocyte, the decline became significant as early as the 2-cell stage for *PCCB*, the 3–4 cell stage for *HSPA1A/L and MON1B*, the 9–16 cell stage for *SLC18A2*, the morula stage for *PMM2*, *TBC1D24*, and *TTLL3*, and the blastocyst stage for *IRF9*. Transcript abundance increased during development only for *PARM1* and *WBP1*. For *PARM1*, there was a slight increase in transcript abundance from the oocyte to 2-cell stage, a large increase at the 9–16 cell and morula stages and then a decline for the blastocyst-stage embryo. For *WBP1*, expression was constant from through the 5-8-cell stage and then increased at the 9–16 cell stage; thereafter amounts declined slightly but non-significantly.

### Protein structure of PARM1 and WBP1

**Fig 2:**
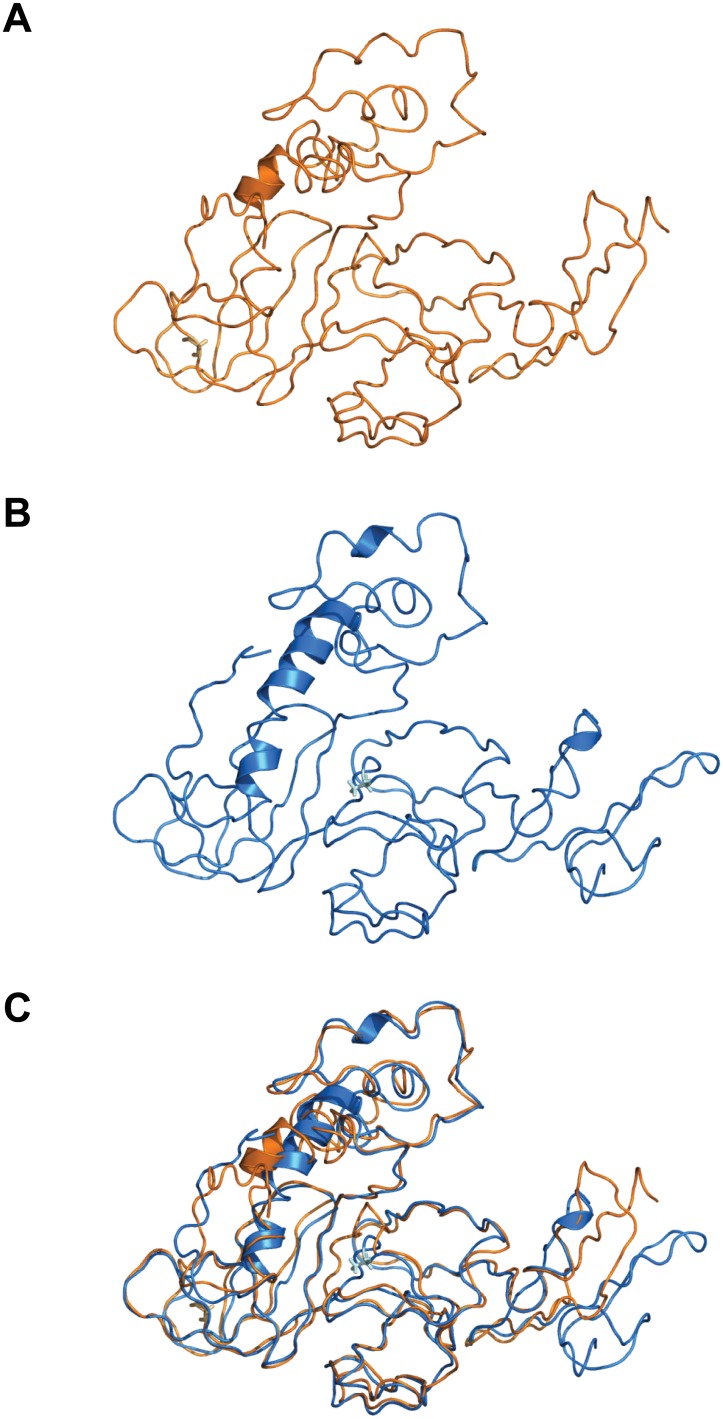
Models of the three-dimensional structure of PARM1. **A**: variant G, corresponding to the major allele; **B**: variant A corresponding to the minor allele; **C**: Superimposed models of both isoforms of the protein; In each panel, the light color side chains represent the residue affected by the SNP.

**Fig 3:**
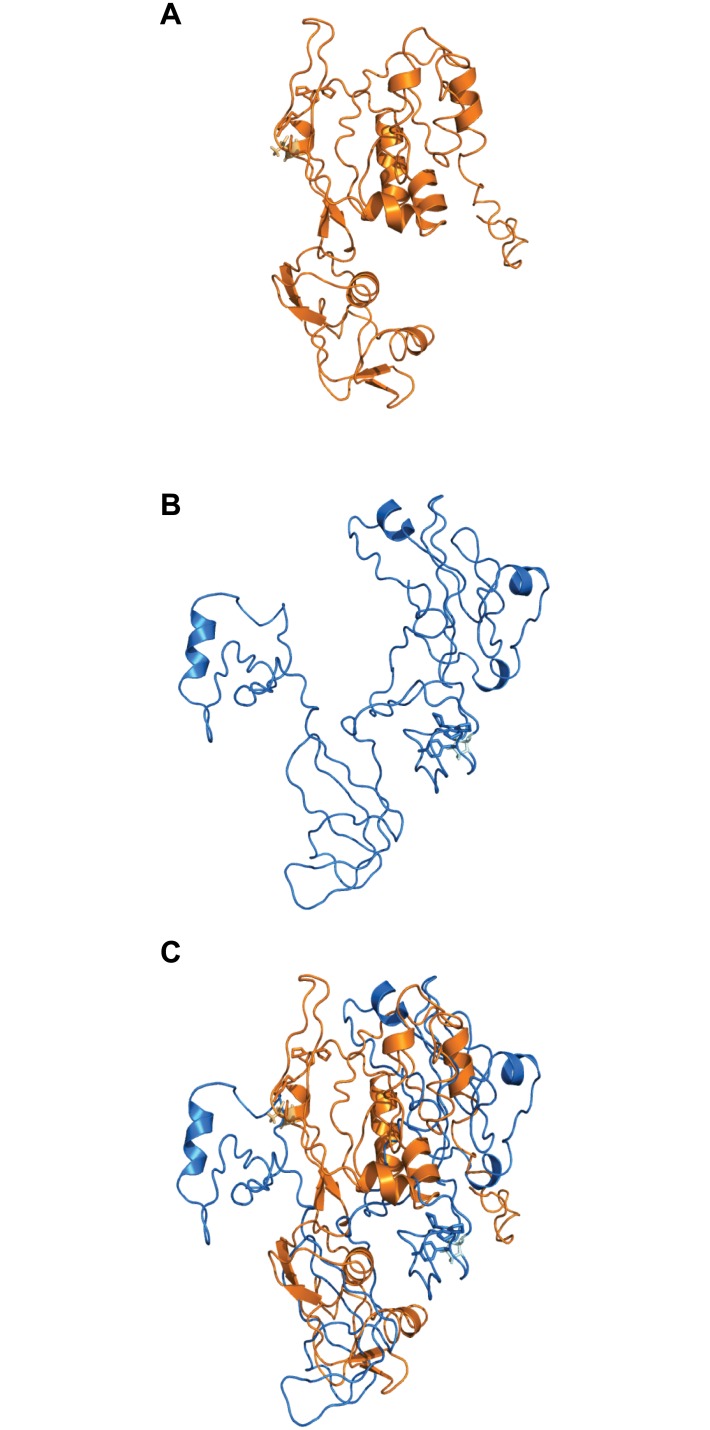
Models of the three-dimensional structure of WBP1. **A**: variant T, corresponding to the major allele; **B**: variant P corresponding to the minor allele; **C**: Superimposed models of both isoforms of the protein. In each panel, the light color side chains represent the position of the AA affected by the SNP.

The two genes in which expression increased coincident with embryonic gene activation were *PARM1* and *WBP1* (Fig 1). To determine whether the amino acid substitution associated with the SNP in these genes could be associated with potential changes in protein conformation/structure, the protein structure prediction algorithm I-TASSER was utilized to generate three-dimensional models of the both variants of PARM1 and WBP1. For PARM1, there were some differences in the superposed alignment between the models of the major and minor allele variants (Fig 2) although the general predicted folding scheme was mostly conserved, with a calculated root mean square deviation (RMSD) of 3.02 Å. Conversely, the variants for WBP1 showed large differences in the predicted protein structure (Fig 3), as the single amino-acid difference shifts the folding scheme such that the generated outputs for both variants cannot be properly aligned (calculated RMSD of 24.44 Å).

### Phylogenetic analysis of the SNP in *WBP1*

**Fig 4:**
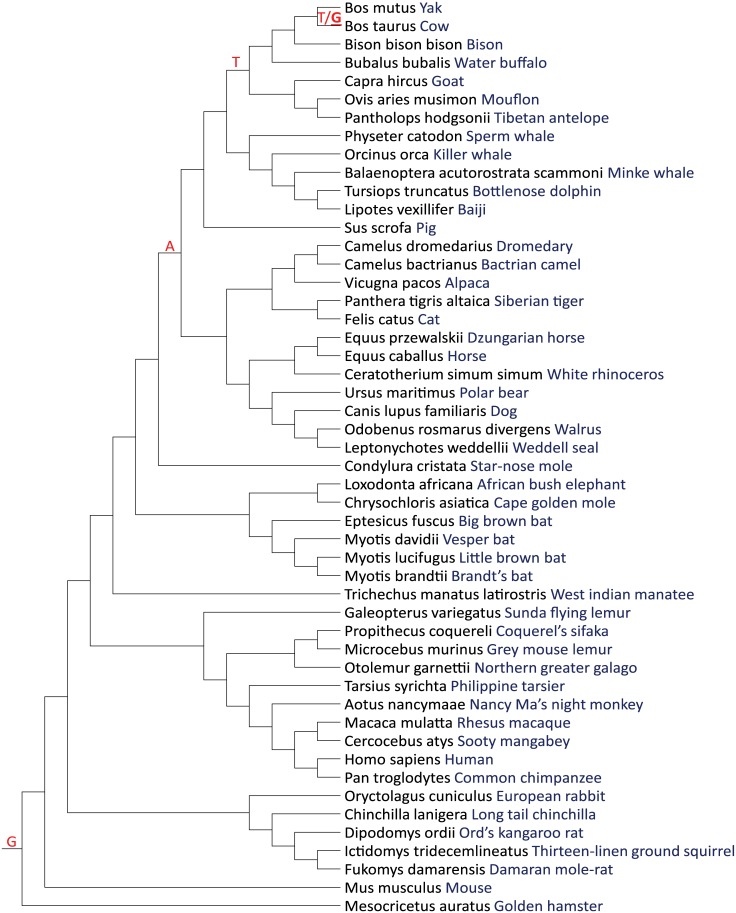
Phylogenetic tree of *WBP1*. The reference allele is indicated in red. All species distal to the common ancestor had the same reference allele unless indicated by placement of a distinct letter. For *B*. *taurus*, both alleles are presented (major/minor). The G allele (underlined) was previously associated with superior development to the blastocyst stage.

For WBP1, P-P-X-Y motifs represent the WW binding domains responsible for protein-protein interactions [20, 38]. As shown in Table 1, the WW P-P-X-Y motifs are conserved in mammals. The SNP in bovine WBP1 is located at 7 positions to the C-terminal to the first amino acid of the second P-P-X-Y motif. Moreover, the amino acid 7 positions from the first amino acid shows significant variation among species (Table 1). To further understand the evolution of variation at this position, the identity of the nucleotide for the SNP in bovine *WBP1* was determined for other species (Fig 4). The reference nucleotide corresponding to common ancestor of carnivores, perrisodactyls, cetartiodactyls, and pigs was either A or T. In cattle, T remains the major allele but the minor allele is G, identical to the reference allele for mammals prior to the common ancestor of carnivores, perrisodactyls, cetartiodactyls, and pigs (Fig 4). Thus, there has been significant genetic change in the nucleotide sequence of *WBP1* at this locus.

**Table 1:** Characteristics of the SNP in *WBP1* associated with embryonic development.

Common name	Scientific name	Position of the amino acid substitution in WBP1 sequence^1^																									
																				^2^0	1	2	3	4	5	6	7
Cow	*Bos taurus*	S	A	F	K	**P**	**P**	**A**	**Y**	E	D	V	V	H	R	P	G	T	P	**P**	**P**	**P**	**Y**	T	A	A	**(T/P)**^3^
Bison	*Bison bison bison*	S	A	F	K	**P**	**P**	**A**	**Y**	E	D	V	V	H	R	P	G	T	P	**P**	**P**	**P**	**Y**	T	A	A	**T**
Pig	*Sus scrofa*	S	A	F	K	**P**	**P**	**A**	**Y**	E	D	V	V	H	R	P	G	T	P	**P**	**P**	**P**	**Y**	T	A	A	**S**
Dog	*Canis lupus*	S	A	F	K	**P**	**P**	**A**	**Y**	E	D	V	V	H	R	P	G	T	P	**P**	**P**	**P**	**Y**	T	A	A	**S**
Vesper bat	*Myotis davidii*	S	A	F	K	**P**	**P**	**A**	**Y**	E	D	V	V	H	R	P	G	T	P	**P**	**P**	**P**	**Y**	T	A	A	**P**
Mouse	*Mus musculus*	S	A	F	K	**P**	**P**	**A**	**Y**	E	D	V	V	H	R	P	G	T	P	**P**	**P**	**P**	**Y**	T	A	A	**P**
Star-nose mole	*Condylura cristata*	S	A	F	K	**P**	**P**	**A**	**Y**	E	D	V	V	H	R	P	G	T	P	**P**	**P**	**P**	**Y**	T	A	A	**P**
Human	*Homo sapiens*	S	A	F	K	**P**	**P**	**A**	**Y**	E	D	V	V	H	R	P	G	T	P	**P**	**P**	**P**	**Y**	T	A	A	**P**
Chimpanzee	*Pan troglodytes*	S	A	F	K	**P**	**P**	**A**	**Y**	E	D	V	V	H	R	P	G	T	P	**P**	**P**	**P**	**Y**	T	V	A	**P**

^1^ P-P-X-Y motifs corresponding to WW binding domains are in bold

^2^ Location of amino acid change in bovine WBP1 relative to the second P-P-X-Y motif

^3^ T encoded by major allele and P encoded by minor allele

### Consequences of knockdown of *WBP1* mRNA for development

**Fig 5:**
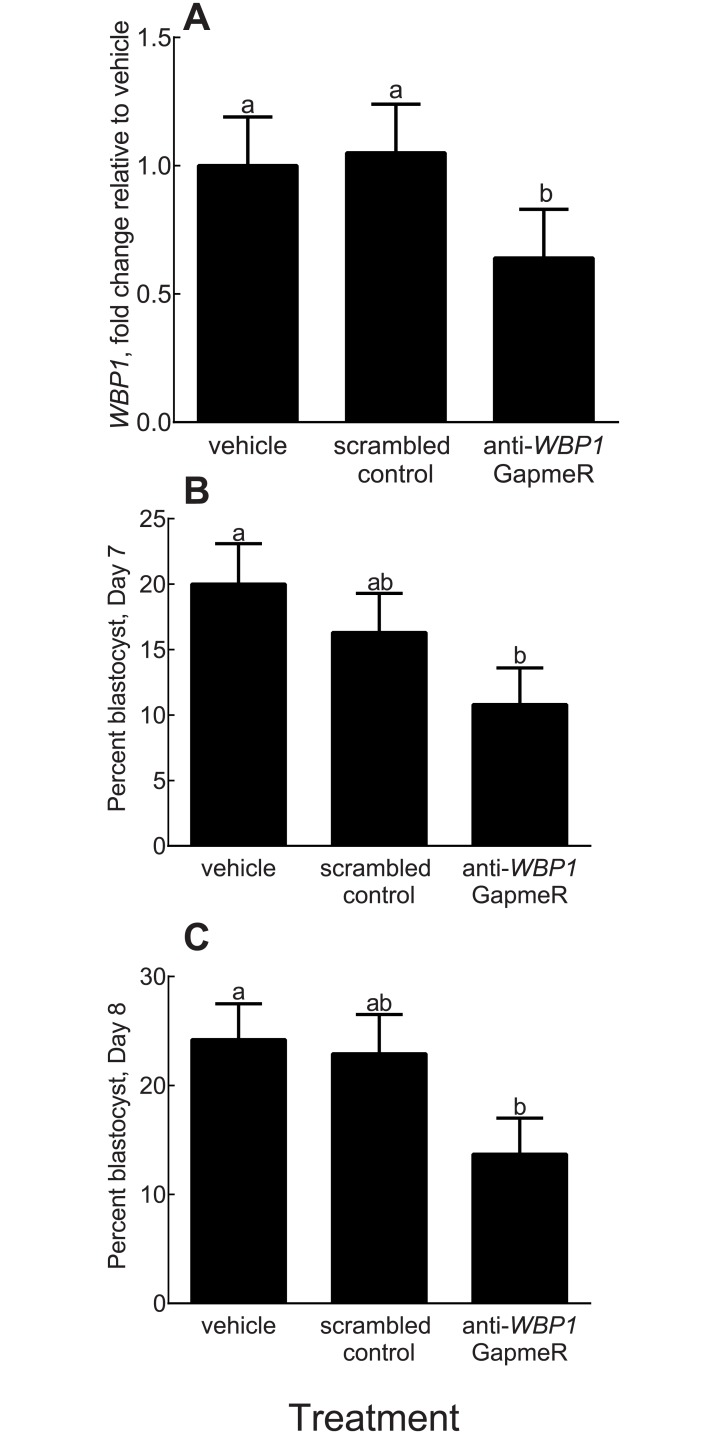
Effects of a *WBP1* antisense oligonucleotide GapmeR on *WBP1* expression and development of the embryo to the blastocyst stage. **A**. Transcript abundance for *WBP1* expression in 9–16 cell preimplantation embryos at 72–76 hpi. Data are least-squares means ± SEM of fold-change values relative to the vehicle treatment. **B** and **C**. Development at Day 7 (B), and Day 8 (C) of development. Data are least-squares means ± SEM of percent of putative zygotes that reached the blastocyst stage.

GapmeR treatment was effective in reducing transcript abundance for *WBP1* because the amount of mRNA for 9–16 cell embryos at 72–75 hpi was reduced for those treated with the anti-WBP1 GapmeR as compared to embryos treated with either the scrambled GapmeR or vehicle (Fig 5A; P = 0.04). There was no difference in expression between embryos treated with the scrambled GapmeR or vehicle.

**Fig 6:**
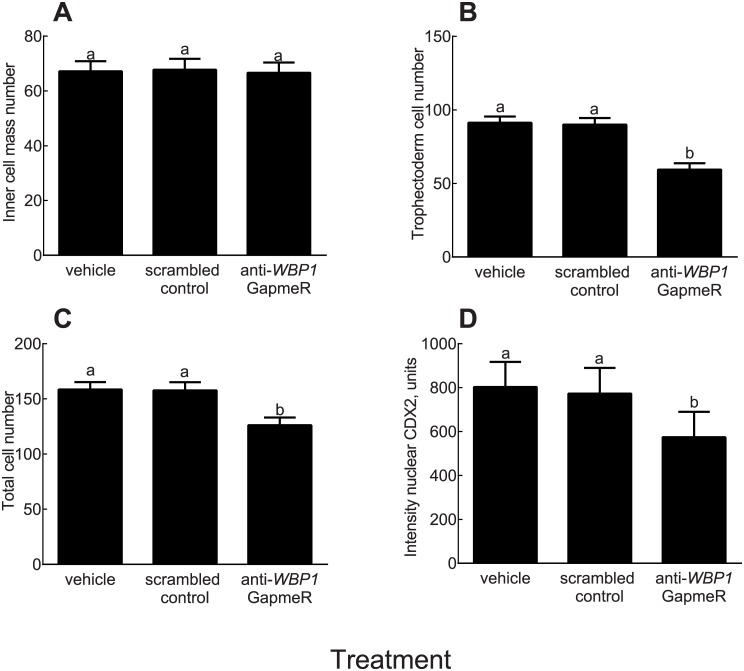
Effects of a *WBP1* antisense oligonucleotide GapmeR on total cell number and intensity of immunoreactive nuclear CDX2 in the bovine blastocyst. **A**. Inner cell mass number **B.** trophectoderm cell number and **C**. Total cell number in blastocyst at Day 8 after insemination. Data are least-squares means ± SEM of cell number. **D.** Immunolocalization of CDX2 measured by net intensity. Data are LSM ± SEM of arbitrary intensity units. In each panel means with different superscripts differ from each other (P<0.05).

**Fig 7:**
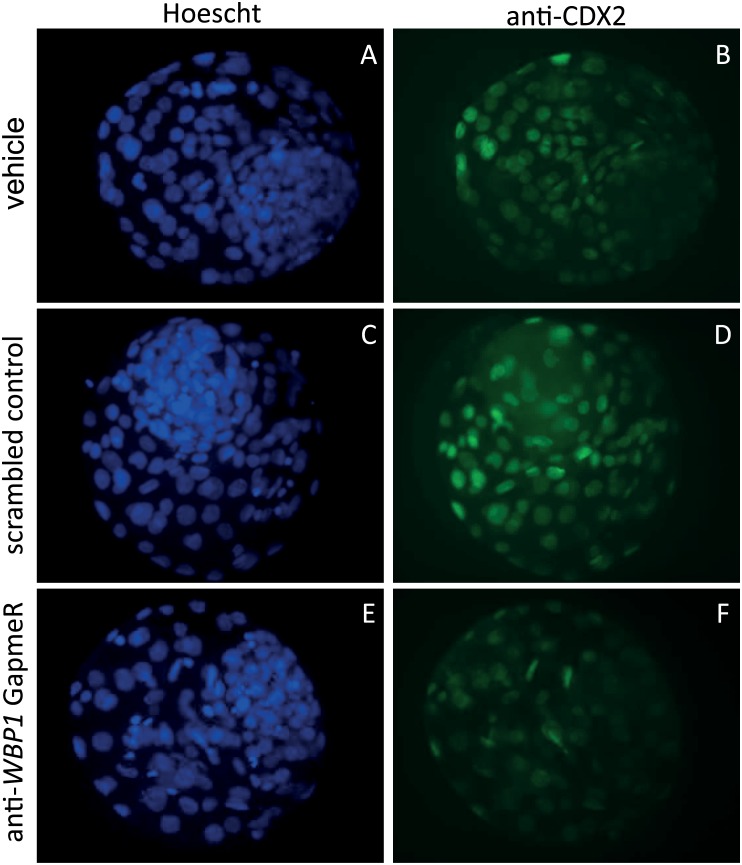
Representative images of labeling of inner cell mass and trophectoderm cells. **A,B.** Vehicle; **C,D**. Scrambled control; **E,F**. anti-*WBP1* GapmeR. Blue (**A,C,E**): nuclei stained with Hoescht 33342. Green (**B,D,F**): trophectoderm cells labeled with antibody against CDX2.

The percent of embryos that cleaved was not affected by treatment (P>0.05; results not shown). However, the percent of putative zygotes that became blastocysts at Day 7 was lower (P = 0.04) for embryos treated with anti-*WBP1* GapmeR compared to embryos treated with the vehicle (Fig 5B). At Day 8, the anti-*WBP1* GapmeR reduced the percent of putative zygotes becoming blastocysts as compared to embryos treated with vehicle (P = 0.04) or scrambled GapmeR (P = 0.08) (Fig 5C). At Day 8, there was no effect of treatment on number of ICM cells in blastocysts (P = 0.83) (Fig 6A) but blastocysts formed after culture with the anti-*WBP1* GapmeR had fewer TE cells (P <0.0001) and fewer total cells (P = 0.0004) than blastocysts formed from embryos treated with the scrambled GapmeR or vehicle (Fig 6B and 6C). Intensity of CDX2 was lower for blastocysts derived from embryos treated with anti-*WBP1* GapmeR (P = 0.0087) than for blastocysts from the two control groups but there was no difference in intensity between the scrambled GapmeR and vehicle (Fig 6D). Representative images of blastocysts are shown in Fig 7.

## Discussion

The overall goal of the present experiment was to understand the possible role of genes containing SNP associated with competence of an embryo to become a blastocyst [15] on preimplantation development. Two of the 12 genes studied, *BRINP3* and *C1QB*, are unlikely to act directly to affect embryonic development because transcripts for the genes were undetectable in the oocyte and embryo. In contrast, evidence was accumulated to indicate that two other genes, *PARM1* and *WBP1*, function in the embryo after embryonic genome activation and during the critical period in development when the embryo undergoes the first differentiation event to form the blastocyst. Moreover, experimental evidence was obtained to demonstrate an important role of WBP1 in development to the blastocyst stage and TE formation.

The only two genes in which there was clear evidence for transcription after embryonic genome was for *PARM1* and *WBP1*. Transcript abundance for both these genes increased at the 9–16 cell stage, a time coincident with the major round of genome activation at the 8-cell stage of development [2]. There was also a smaller increase in expression of *PARM1* at the 2-cell stage. Thereafter, steady-state mRNA for *PARM1* continued to increase to the morula stage of development, when the embryo forms junctional complexes between outer cells in preparation for blastocyst formation, before declining in the blastocyst, when the embryo first differentiates into inner cell mass and trophectoderm. Transcript abundance for *WBP1* peaked at the 9–16 cell stage and experienced a small decline thereafter.

The roles of PARM1 and WBP1 in the preimplantation embryo are not known but both exert cellular functions that are potentially important for development during the time the genes are expressed. PARM1 is a mucin-like type 1 transmembrane protein that can inhibit apoptosis [39] and promote differentiation of cardiomyocytes by increasing mRNA for *BMP2* and *BMP4* [40]. Bone morphogenic proteins have been implicated in promoting differentiation of the bovine embryo. Culture of embryos with BMP2 increased expression of the trophoblast transcription factor *CDX2* and the pluripotency factor *NANOG* [41], while BMP4 decreased expression of the pluripotency factor *POU5FI* [42], and increased formation of trophoblastic cell lines from bovine blastocysts [43]. Polymorphisms in *BMP4* have been associated with development of bovine embryos to the blastocyst stage [14]). WBP1 is a single transmembrane adaptor protein [44] that functions to bind to a variety of signaling proteins containing WW1 or WW2 domains. Among these are the proteins KIBRA, SAV1, and YAP involved in the Hippo signaling pathway [45]. Hippo signaling has been implicated in differentiation of the blastocyst in the mouse [21, 46]. The transcription factor YAP interacts with TEAD4 to induce transcription of *CDX2* which in turn causes differentiation of the outer cells of the developing blastocyst into trophectoderm [21].

Experimental evidence was obtained that WBP1 plays an important role in embryonic development. In particular, reduction in transcript abundance for *WBP1* using RNAi technology caused a reduction in number of putative zygotes becoming a blastocyst and reduced the number of TE cells in those embryos becoming blastocysts. It is likely that *WBP1* reduced TE formation through its involvement in accumulation of CDX2 in the nucleus because GapmeR treatment reduced intensity of immunoreactive CDX2 in the nucleus.

The role of *HSPA1L*, *IRF9*, *MON1B*, *PCCB*, *PMM2*, *SLC18A2*, *TBC1D24*, and *TTLL3* in embryonic development were not resolved in the current study. Transcript abundance for all of these genes declined as the embryo advanced in development. Such a decline probably reflects the large-scale destruction of maternally-derived mRNA stored in the oocyte that occurs after fertilization [6, 47]. Moreover, none of these genes experienced an increase in transcript abundance at the 9–16 cell stage. One interpretation of this finding is that the genes are not transcribed during the period of embryonic development. This is not necessarily true, however, since it is possible that contributions of newly-synthesized embryonic mRNA were masked by the degradation of maternal mRNA. Using detection of intronic sequences by RNA-Seq as evidence of transcription, Graf *et al*. [17] found that *TTLL3* was first transcribed at the 16-cell stage and *PCCB* and *TBC1D24* at the blastocyst stage. However, there was no evidence for transcription of *IRF9*, *MON1B*, *PMM2*, *SLC18A2*, or *TBC1D24* in that study. It remains possible that the association between SNP in these genes and embryonic development reflect either an indirect effect on sperm used for fertilization, linkage with a causative SNP in another gene, or that the original association was incorrect.

The situation for *HSPA1L*, which is the only gene studied in which a SNP in the promoter has been identified, is more complex. This gene is nearly identical to *HSPA1A* so that primers used for RT-PCR, including those used here, do not distinguish between the genes. Transcription of *HSPA1A/L* does occur in the bovine embryo in response to heat shock as early as the two-cell stage [48], and there is presence of the protein in the embryo from the 2-cell stage through ≥16-cell stage [49]. Therefore, it is possible that paternally-derived genes could influence embryonic development. Moreover, there is evidence from both lymphocytes [16] and the preimplantation embryo [50] that the deletion mutation in the promoter of *HSPA1L* is associated with increased resistance of cells to heat shock. Thus, the gene could be important in situations in which the embryo experiences cellular stress.

Although results indicate that both *PARM1* and *WBP1* are transcribed after embryonic genome activation, and that *WBP1* is important for TE formation, consequences of the SNP in these genes for embryonic development remain to be resolved. Based on structural predictions, the SNP in *PARM1* causes subtle differences in the predicted tertiary structure but whether these differences are sufficient to change protein function is not known. For *WBP1*, in contrast, the effects of the SNP are pronounced. First, the SNP causing a threonine to be replaced with proline would be expected to markedly change the shape of the protein, as proline residues are associated with turns in folded proteins [51]. Indeed, the prediction algorithm generates a large difference in the folding pattern between WBP1 variants. Furthermore, the mutation in *WBP1* causes an amino acid substitution at a residue close in proximity to a P-P-X-Y binding motif. The P-P-X-Y motif interacts with other proteins/peptide-ligands, and changes to nearby residues can either impact the accessibility of this region by potentially enhancing or limiting the motif-based functionality of WBP1 [20, 38]. Additional characterization of these proteins via structural biology techniques (e.g. x-ray crystallography) will provide major insights into the accuracy of the prediction models as well as elucidate regions of possible functional importance.

Phylogenetic analysis of the nucleotide corresponding to the SNP in bovine *WBP1* encoding for the amino acid at the position 7 downstream from one of the P-P-X-Y motifs has been subject to variation among and within species. Presence of proline residues in flanking regions of the P-P-X-Y motif has been predicted to increase the tightness of binding of the motif to WW domains [20]. While mutations at position 7 have not been examined, amino acid substitutions at the amino acid in positions 4, 5, or 6 can affect binding of human YAP to the P-P-X-Y motif [38]. The fact that the nucleotide encoding the amino acid at this position is not conserved suggests that sequence of *WBP1* at this locus may have been subjected to natural selection.

In summary, results indicate that of 12 genes previously found associated with genetic variation in embryonic development, two (*PARM1* and *WBP1)*, are likely to function in the embryo after embryonic genome activation because transcript abundance increases at the 9–16 cell stage. Two other genes (*BRINP3* and *C1QB*) are unlikely to directly affect embryonic development because transcripts were undetectable in the oocyte and embryo. The role of the other 8 genes (*HSPA1L*, *IRF9*, *MON1B*, *PCCB*, *PMM2*, *SLC18A2*, *TBC1D24*, and *TTLL3)* is unclear because, while mRNA was present, transcript abundance declined as the embryo advanced in development. A critical role for WBP1 in formation of the TE was demonstrated by reducing transcript abundance using an antisense GapmeR designed against *WBP1*. Moreover, the SNP in *WBP1* previously associated with embryonic development is located near one of the P-P-X-Y motifs required for protein-protein binding and causes a large change in predicted protein structure. The locus corresponding to the SNP in bovine *WBP1* has been subject to genetic selection in mammals. It was concluded that *WBP1* is an important gene for embryonic development in the cow. Further research to identify how the SNP in *WBP1* affects processes leading to differentiation of the embryo into TE and ICM lineages is warranted.

## Supporting information

S1 File Raw data used for the paper. (XLSX)

S1 Table Composition of media used for *in vitro* production of embryos. (DOCX)

S2 Table Primers used for quantitative RT-PCR. (DOCX)
