# Supplementary material for: Characteristics of candidate genes associated with embryonic development in the cow: Evidence for a role for WBP1 in development to the blastocyst stage
Source: PLoS One. 2017 May 18;12(5):e0178041. doi: 10.1371/journal.pone.0178041 (PMC5436885; doi:10.1371/journal.pone.0178041)
Supplement: S1 Table — (DOCX) [file pone.0178041.s002.docx]

**S1 Table**. Composition of media used for *in vitro* production of embryos.^a^

| Medium | Ingredient | Concentration |
| --- | --- | --- |
| OCM | Tissue culture medium 199 (Hank’s salts, L-glutamine and without bicarbonate)^b^ |  |
|  | NaHCO_3_ | 4.16 mM |
|  | HEPES | 9.98 mM |
|  | Alanyl-glutamine | 1 mM |
|  | Bovine steer serum | 2% v/v |
|  | Heparin | 2 U/ml |
|  | Penicillin | 100 U/ml |
|  | Streptomycin | 0.1 mg/ml |
|  |  |  |
| OMM | Tissue culture medium 199 (with Earle’s salts)^b^ |  |
|  | Bovine steer serum | 10% v/v |
|  | Estradiol-17β | 2 µg/ml |
|  | Follicle stimulating hormone | 20 µg/ml |
|  | Sodium pyruvate | 22 µg/ml |
|  | Gentamicin | 50 µg/ml |
|  | Alanyl-glutamine | 1 mM |
|  |  |  |
| HEPES-SOF | HEPES | 10 mM |
|  | CaCl_2_•2H_2_O | 1.17 mM |
|  | MgCl_2_•6H_2_O | 0.49 mM |
|  | KH_2_PO_4_ | 1.19 mM |
|  | KCl | 7.16 mM |
|  | NaCl | 107.7 mM |
|  | NaHCO_3_ | 2.0 mM |
|  | Na-lactate | 5.3 mM |
|  | BSA, fraction V | 45.2 mM |
|  | Sodium pyruvate | 20 mM |
|  | Gentamicin | 7.5 µg/ml |

**S1 Table (continued)**. Composition of media composition used for *in vitro* production of embryos.

| Medium | Ingredient | Concentration |
| --- | --- | --- |
| SOF-FERT | CaCl_2_•2H_2_0 | 1.17 mM |
|  | MgCl_2_•6H_2_O | 0.49 mM |
|  | KH_2_PO_4_ | 1.19 mM |
|  | KCl | 7.16 mM |
|  | NaCl | 107.7 mM |
|  | NaHCO_3_ | 25.07 mM |
|  | Na-lactate | 5.3 mM |
|  | Essentially fatty acid free BSA | 60 mg/ml |
|  | Sodium pyruvate | 0.2 mM |
|  | Gentamicin | 5 µg/ml |
|  | Heparin | 10 µg/ml |
|  | Caffeine | 194.2 µg/ml |
|  |  |  |
| PHE^c^ | Hypotaurine | 0.25 mM |
|  | Penicillamine | 0.50 mM |
|  | Epinephrine | 25 µM |
|  |  |  |
| SOF-BE2 | NaCl | 92 mM |
|  | KCl | 6.02 mM |
|  | KH_2_PO_4_ | 1 mM |
|  | CaCl_2_•2H_2_O | 0.98 mM |
|  | Sodium lactate | 4.5 mM |
|  | NaHCO_3_ | 21.06 mM |
|  | MgCl_2_•6H_2_O | 0.41 mM |
|  | Alanyl-glutamine | 0.93 mM |
|  | Sodium pyruvate | 0.37 mM |
|  | Trisodium citrate | 0.4 mM |
|  | Myo-inositol | 2.56 mM |
|  | Gentamicin sulfate | 23.3 mM |
|  | Non-essential amino acids (100X) | 9.3 µl/ml |
|  | Essential amino acids (50X) | 18.6 µl/ml |
|  | Essentially fatty acid free BSA | 3.7 mg/ml |

^a^ Abbreviations are as follows: OCM, oocyte collection medium; OMM, oocyte maturation medium; HEPES-SOF, HEPES-buffered synthetic oviduct fluid; SOF-FERT, synthetic oviduct fluid for fertilization; PHE, penicillamine-hypotaurine-epinephrine solution; SOF-BE2, synthetic oviduct fluid-bovine embryo 2.

^b^ All other ingredients were added to 1 L of tissue culture medium 199. The final concentrations listed in the table account for the dilution resulting from addition of the ingredients.

^c^ Prepare, as fresh solutions, primary stocks of 1 mM hypotaurine, 2 mM penicillamine and 250 µM epinephrine (prepared by dissolving in a lactate-metabisulfite solution). The PHE is prepared by combining 10 ml 1 mM hypotaurine, 10 ml 2 mM penicillamine, 4 ml of 250 µM epinephrine and 16 ml of 0.9% (W/v) NaCl. Note that the lactate-metabisulfite solution is prepared by adding 77 µl of a 98% (w/w) sodium lactate syrup and 50 mg sodium metabisulfite to 50 ml water.
