# Supplementary material for: Characteristics of candidate genes associated with embryonic development in the cow: Evidence for a role for WBP1 in development to the blastocyst stage
Source: PLoS One. 2017 May 18;12(5):e0178041. doi: 10.1371/journal.pone.0178041 (PMC5436885; doi:10.1371/journal.pone.0178041)
Supplement: S2 Table — (DOCX) [file pone.0178041.s003.docx]

**S2 Table.** Primers used for quantitative RT-PCR.

| rs number | Gene symbol | Sequence accession ID | Primer sequences | bp |
| --- | --- | --- | --- | --- |
| rs135390325 | *C1QB* | NM_001046599.2 | F:5’-CGC TTC GAC CAC GTT ATC A-3’ | 101 |
|  |  |  | R:5’-GAGATGAAGAAGTGGATGGTACG-3’ |  |
| rs135071345 | *BRINP3* | NM_001102140.2 | F:5’-CCG CTA TGA ACA ACT GGA GAA-3’ | 92 |
|  |  |  | R:5’-AAATCGGAATCGTTCTCCACAG-3’ |  |
| - | *HSPA1L** | XM_005223678.1 | F: 5’-GGGGAGGACTTCGACAACAGG-3’ | 245 |
|  |  |  | R: 5’-CGGAACAGGTCGGAGCACAGC-3’ |  |
| rs110953315 | *IRF9* | NM_001024506.1 | F:5’-GAG GAA GAA GTT ACC GCA AAG A-3’ | 118 |
|  |  |  | R:5’-AAAACTTGAAACCCTCGTGGC-3’ |  |
| rs41859871 | *MON1B* | NM_001037454.2 | F:5’-CTG ACC TTC CTA CAC ACT CTT TC-3’ | 94 |
|  |  |  | R:5’-GATACGTCCACTGTTGTCACT-3’ |  |
| rs111027720 | *PARM 1* | NM_001075771.2 | F:5’-AGC TCT ACT CTC ACA CCT ACT T-3’ | 119 |
|  |  |  | R:5’-CTATCTGTACCTCTGGTGGTG-3’ |  |
| rs109813896 | *PCCB* | NM_001038548.2 | F:5’-TCA CTT TGA GCT GAC CCT TAT C-3’ | 102 |
|  |  |  | R:5’-TTATTCGGAAATCCCACTTGATGA-3’ |  |
| rs109629628 | *PMM2* | NM_001035095.2 | F:5’-GGC TTG GTA GCA TAC AGA GAT G-3’ | 109 |
|  |  |  | R:5’-GTTGATGACAGACTCGATGTAGC-3’ |  |
| rs110365063 | *SLC18A2* | NM_174653.2 | F:5’-CAG CCC TTC ATG TCT CTG ATT-3’ | 112 |
|  |  |  | R:5’-TACA T TCACCTGGGTACGTC-3’ |  |
| rs110660625 | *TBC1D24* | NM_001046296.2 | F:5’-TGG AGA GTG CTT CGT GTT TAG-3’ | 77 |
|  |  |  | R:5’-GTAGTTTGTGGGTCTCGACT-3’ |  |
| rs135236119 | *TTLL3* | XM_010817598.1 | F:5’-TTT GAT TTC CCT CCC ACC TAA C-3’ | 122 |
|  |  |  | R:5’-TTCATTACACACCAGAGTCGG-3’ |  |
| rs134282928 | *WBP1* | NM_001034346.1 | F:5’-CTG ACT GGT GAC TCA GGT ATT G-3’ | 81 |
|  |  |  | R:5’-AGTTCCTCCGATCCCAATCA-3’ |  |

*Primers amplify both *HSPA1A* and *HSPA1L*
